# Quality of life in Turkish children with interstitial lung disease: Insights from the National chILD Registry

**DOI:** 10.1007/s00431-026-06834-5

**Published:** 2026-04-01

**Authors:** Handan Kekec, Tugba Sismanlar Eyuboglu, Ayse Tana Aslan, Ozge Ulgen, Aslıhan Ozcaglar, Zeliha Başak Polat, Betül Bankoglu Parlak, Aysen Bingol, Seyda Karabulut, Ela Erdem Eralp, Cigdem Korkmaz, Berrak Oztosun, Gokcen Kartal Ozturk, Bahar Girgin Dindar, Meltem Akgul Erdal, Umay Kavgacı, Esin Gizem Olgun, Fatma Nur Ayman, Zeynep Seda Uyan, Beste Ozsezen, Tuğba Ramaslı Gursoy, Pelin Asfuroglu, Mahir Serbes, Mina Hızal, Halime Nayır Buyuksahın, Gözde Cavıldak Karaaslan, Ebru Yalcın, Nagehan Emiralioglu, Yasemin Gökdemir, Sevgi Pekcan, Ayşe Ayzıt Kılınc Sakallı, Saniye Girit, Nazan Cobanoglu, Güzin Cinel, Berna Oguz, Diclehan Orhan, Ahmet Cevdet Ceylan, Nural Kiper

**Affiliations:** 1 Department of Pediatric Pulmonology, Faculty of Medicine, Gazi University, Ankara, Turkey; 2 Department of Pediatric Pulmonology, Faculty of Medicine, Medeniyet University, Istanbul, Turkey; 3 Department of Pediatric Pulmonology, Faculty of Medicine, Akdeniz University, Antalya, Turkey; 4 Department of Pediatric Pulmonology, Faculty of Medicine, Marmara University, Istanbul, Turkey; 5 Department of Pediatric Pulmonology, Cerrahpasa Faculty of Medicine, Istanbul University-Cerrahpasa, Istanbul, Turkey; 6 Department of Pediatric Pulmonology, Behcet Uz Research and Training Hospital, Izmır, Turkey; 7 Department of Pediatric Pulmonology, Faculty of Medicine, Ege University, Ankara, Turkey; 8 Department of Pediatric Pulmonology, Faculty of Medicine, Hacettepe University, Ankara, Turkey; 9 Department of Pediatric Pulmonology, Faculty of Medicine, Ankara University, Ankara, Turkey; 10 Department of Pediatric Pulmonology, Faculty of Medicine, Necmettin Erbakan University, Konya, Turkey; 11 Department of Pediatric Pulmonology, Faculty of Medicine, Koç University, Istanbul, Turkey; 12 Department of Pediatric Pulmonology, Faculty of Medicine, Dokuz Eylül University, Izmir, Turkey; 13 Department of Pediatric Pulmonology, Van Research and Training Hospital, Van, Turkey; 14 Department of Pediatric Pulmonology, Cengiz Gökçek Research and Training Hospital, Gaziantep, Turkey; 15 Department of Pediatric Allergy and Immunology, Faculty of Medicine, Çukurova University, Adana, Turkey; 16 Department of Pediatric Pulmonology, Ankara Research and Training Hospital, Ankara, Turkey; 17 Department of Pediatric Pulmonology, Mardin Research and Training Hospital, Mardin, Turkey; 18 Department of Pediatric Pulmonology, Faculty of Medicine, Medipol University, Istanbul, Turkey; 19 Department of Pediatric Pulmonology, Bilkent City Hospital, Ankara, Turkey; 20 Department of Radiology, Faculty of Medicine, Hacettepe University, Ankara, Turkey; 21 Department of Pathology, Faculty of Medicine, Hacettepe University, Ankara, Turkey; 22 Department of Genetics, Bilkent City Hospital, Ankara, Turkey

**Keywords:** Childhood interstitial lung disease, chILD, Pediatric quality of life, PedsQL, Fan score, Lung function

## Abstract

Childhood interstitial lung diseases (chILD) include a diverse range of rare disorders, complex clinical progressions, and high rates of morbidity and mortality. This study aims to assess the clinical and psychosocial impacts of chILD in children registered in the National chILD-Turkiye database. Data from 17 centers were analyzed as of January 2024. Patients aged 1 month to 18 years were included in the study. Through face-to-face interviews, patients and their families completed the Pediatric Quality of Life Inventory (PedsQL™ 4.0 Generic Core Scales) and PedsQL™ 2.0 Family Impact Module. Higher scores suggest better performance and higher health-related quality of life (HRQoL). Patient clinic data, demographic information, pulmonary function tests, Fan scores, and household income were recorded. Patients were categorized into two groups according to their use of O_2_ therapy and diffuse progressive lung disease (DPLD) groups A and B. The study included 175 patients (90 girls), with a median age of 7.7 (1.0–17.8) years. A strong positive correlation was found between the FEV1% value and both the Physical Health Summary Score and the Total Score (*p* = 0.007, *r* = 0.608; *p* = 0.008, *r* = 0.607). The Fan score negatively correlated with PedsQL™ scores (*p* < 0.001). Patients who use O_2_ have an earlier diagnosis of age of chILD, lower FEV1%, higher Fan scores, and lower Child Self-Reports and Parent Reports scores (*p* < 0.05). The group with DPLD-A disorders has an earlier diagnosis age, lower weight and BMI z-scores, higher Fan score, and higher Family Impact Module score (*p* < 0.05).

*Conclusion*: Lung function and Fan score strongly correlate with HRQoL, emphasizing the impact of disease severity on physical and psychosocial well-being. These findings highlight the need for multidisciplinary care and psychosocial support to enhance the QoL in children with chILD and their families.

**Table:** 

**What is Known:** •*Childhood interstitial lung diseases (chILD) are rare and diverse disorders associated with significant morbidity and complex management.* •*Evidence regarding health-related quality of life (HRQoL) and the psychosocial burden on affected families remains limited.*
**What is New:** •*This multicenter national registry–based study provides the first comprehensive evaluation of HRQoL and family impact in children with chILD in Türkiye.* •*Lung function impairment and higher Fan severity scores were strongly linked to poorerHRQoL, highlighting the influence of disease severity on physical and psychosocial well-being.*

**What is Known:**

•*Childhood interstitial lung diseases (chILD) are rare and diverse disorders associated with significant morbidity and complex management.*

•*Evidence regarding health-related quality of life (HRQoL) and the psychosocial burden on affected families remains limited.*

**What is New:**

•*This multicenter national registry–based study provides the first comprehensive evaluation of HRQoL and family impact in children with chILD in Türkiye.*

•*Lung function impairment and higher Fan severity scores were strongly linked to poorerHRQoL, highlighting the influence of disease severity on physical and psychosocial well-being.*

## Introduction

Childhood interstitial lung diseases (chILD) include over 200 rare disorders known for their complex clinical progression, as well as high morbidity and mortality rates. The mortality rate is reported to be around 15% [1, 2]. ChILD can present a severe clinical course; children may experience hypoxemia and require long-term oxygen therapy or ventilatory support, including non-invasive (NIV) or invasive mechanical ventilation (MV). Children with chronic lung diseases often face nutritional deficiencies and growth impairments due to increased energy needs, decreased appetite, and ongoing inflammation, indicating that nutritional support may be essential for patients with chILD. Children with chILD often encounter numerous challenges related to their condition. These include a complex diagnostic process, the need for extended medication regimens, regular visits to outpatient clinics or emergency departments, frequent hospitalizations, limitations on physical activity, and feelings of social isolation. The chronic and unpredictable nature of the disease heightens anxiety, uncertainty, and emotional exhaustion within caregivers. As a result, the overall quality of life (QoL) for both the child and their family can be significantly affected, impacting not only physical health but also psychological and functional well-being [3–5].

Generative and disease-specific QoL questionnaires have been developed to evaluate these multidimensional effects. The Pediatric Quality of Life Inventory (PedsQL) offers a valuable and comprehensive assessment by integrating child self-reports and parent proxy-reports. This dual perspective captures not only the child’s physical and psychosocial experiences but also the family’s viewpoint. Given the chronic and unpredictable nature of chILD and its significant impact on families, the PedsQL—including its Family Impact Module—offers a sensitive and structured approach for monitoring health outcomes and directing patient-centered care [3, 6–9].

Data assessing the QoL and family impact on children with chILD remain limited. In this study, we aimed to evaluate the clinical, familial, and psychosocial effects of the disease in children diagnosed with chILD in the National chILD-Turkiye (chILD-TR) database.

## Methods

This study was a cross-sectional, multicenter research project using data collected from the chILD-TR. All patients aged 1 month to 18 years, diagnosed with chILD and registered in the chILD-TR database by July 1, 2024, who also provided consent to participate, were included. Patients who were unreachable or declined to participate were excluded. This research was conducted in accordance with the principles of the Declaration of Helsinki. It received approval from Gazi University Ethics Board on 09.07.2024, meeting 12, reference number: 2024–1189. All participants provided written informed consent for their participation and the publication of their data. For participants under 18, consent was obtained from a parent or guardian.

In the CHILD-TR, patient data are collected from multicenter sites across Turkey using patient codes. The demographic information includes gender, consanguinity, current age, age at first complaint, age at first admission, and follow-up duration. Clinical data encompass growth z-scores, symptoms, physical examination findings, and radiological details. Additionally, pulmonary function tests (PFT), diffusing capacity of the lung for carbon monoxide (DLCO), and respiratory support status—such as oxygen therapy via nasal cannula, NIV, and MV—are documented. The number of hospital admissions in the past year, along with the ages of the patient’s mother and father, as well as household income status (categorized as income less than expenditure, equal to expenditure, or exceeding expenditure), is also recorded.

### Classification of chILD diagnosis

The classification system developed by the chILD-EU study group was used to categorize diseases within the national registry, chILD-TR, which was established in November 2021 [10, 11]. In the chILD-EU classification system, diagnoses are grouped into Diffuse Parenchymal Lung Diseases (DPLD) types A and B. DPLD was first published and histologically categorized by Deutsch et al. [12] in 2007 into two main groups: DPLD-A, which includes disorders more common in infancy, and DPLD-B, which encompasses diseases that can occur at any age. In 2015, Griese et al. [13] further expanded this DPLD classification and its subcategories. In the chILD-TR, similar to the chILD-EU, the current subcategories of A-DPLD disorders, mainly manifesting in infancy, include A1, A2, A3, A4, Ax, and Ay. The current subcategories of B-DPLD disorders, which can occur at any age, include B1, B2, B3, B4, B5, and Bx.

### Pulmonary function tests

Pulmonary function tests were performed according to the standards established by the American Thoracic Society/European Respiratory Society (ATS/ERS) guidelines for pediatric patients. Forced Expiratory Volume in 1 s (FEV1), forced vital capacity (FVC), peak expiratory flow (PEF), and forced expiratory flow from 25 to 75% (FEF_25–75_%) were measured and recorded as percentages. The FEV1/FVC ratio was also evaluated considering age, gender, and height [14]. The DLCO was assessed using the single-breath method. Results between the 5th and 95th percentiles were considered within normal limits [15].

### Fan score

The severity-of-illness score, developed by Fan et al., evaluates patients diagnosed with chronic interstitial lung disease (ILD). Based on their symptoms, oxygen requirement, and presence of pulmonary hypertension, patients are assigned scores ranging from 1 to 5. A score of 1 indicated asymptomatic patients; 2 indicated symptomatic patients with normal room air oxygen saturation under all conditions; 3 indicated symptomatic patients with normal resting oxygen saturation but desaturation (≤ 90%) during sleep or exercise; 4 indicated symptomatic patients with abnormal resting room air saturation (≤ 90%); and 5 indicated symptomatic patients with pulmonary hypertension. A higher score signifies a more severe clinical condition [16].

### Pediatric QoL and family impact evaluation [8, 9]

Permission to use the Turkish version of the PedsQL™ 4.0 Generic Core Scales, PedsQL™ Infant Scales, and the Family Impact Module was obtained through a license agreement signed with the Mapi Research Trust.

To evaluate the health-related quality of life (HRQoL) of the patients and their caregivers, the PedsQL™ Generic Core Scales 4.0 and PedsQL™ Infant Scales (standard forms) questionnaire were administered to the patients, while the PedsQL™ 2.0 Family Impact Module (standard form) was completed by family members involved in the care of these children. The questionnaires were conducted in person with all caregivers and, based on age, with the patients themselves during outpatient clinic visits at the participating centers.

The PedsQL QoL questionnaire is categorized by age groups as follows: Young infants are from 1 to 12 months, older infants range from 13 to 24 months, toddlers are between 2 and 4 years, young children fall between 5 and 7 years, children are from 8 to 12 years, and adolescents are aged 13 to 18 years.

For patient-reported outcomes (PRO), children between the ages of 5 and 7 were given a simplified selection of responses to choose from: never (0), sometimes (2), and almost always (4). Children aged 8 years and older were asked to respond to questions regarding their physical, emotional, social, and school functioning during the past month, selecting one of the following response options: never (0), almost never (1), sometimes (2), often (3), and almost always (4). The scores for Psychosocial Health Summary, Physical Health Summary, and the Total Score were computed.

For parent proxy-reports, caregivers were asked to assess their child’s physical, emotional, social, and school functioning over the past month by selecting one of the following response options, 0 to 4. Psychosocial Health Summary Score, Physical Health Summary Score, and the Total Score were calculated.

The PedsQL™ 2.0 Family Impact Module includes eight subscales: physical functioning, emotional functioning, social functioning, cognitive functioning, communication, worry, daily activities, and family relationships. Participants rate these based on their experiences over the past month by choosing one of the following options: never (0), almost never (1), sometimes (2), often (3), or almost always (4). The Parent HRQoL Summary Score, the Family Functioning Summary Score, and the Total Score were calculated.

### Scoring

Based on the questionnaire responses, items are reverse-scored and linearly transformed to a scale ranging from 0 to 100 (0 = 100, 1 = 75, 2 = 50, 3 = 25, 4 = 0). A higher score indicates a better QoL with fewer problems or symptoms.

#### Statistical analysis

Analyses were performed using IBM SPSS Statistics version 22.0 (IBM, Armonk, NY, USA). Data normality was evaluated visually through histograms and probability plots and analytically via the Shapiro–Wilk test. Continuous variables were expressed as means and standard deviations for normal distribution and as medians with ranges for non-normally distributed data. Categorical variables were reported as frequencies and percentages. To compare two independent groups, Student’s *t*-test was applied for normally distributed variables, while the Mann–Whitney *U* test was used for non-normally distributed ones. For within-group comparisons, the paired *t*-test was utilized for normally distributed variables, and the Wilcoxon signed-rank test for non-normally distributed data. Correlation analyses used Pearson’s correlation coefficient for normally distributed data and Spearman’s rank correlation for non-normally distributed data. The strength of the correlation was interpreted as very weak (*r* ≤ 0.2), weak (*r* = 0.2–0.4), moderate (*r* = 0.4–0.6), strong (*r* = 0.6–0.8), or very strong (*r* > 0.8). A *p*-value of less than 0.05 was considered statistically significant.

### Household income status

Socioeconomic data were collected through questionnaires completed by caregivers during the data collection process. Caregivers were asked to indicate whether their monthly household income was less than, equal to, or greater than their monthly expenses.

## Results

**Fig. 1:**
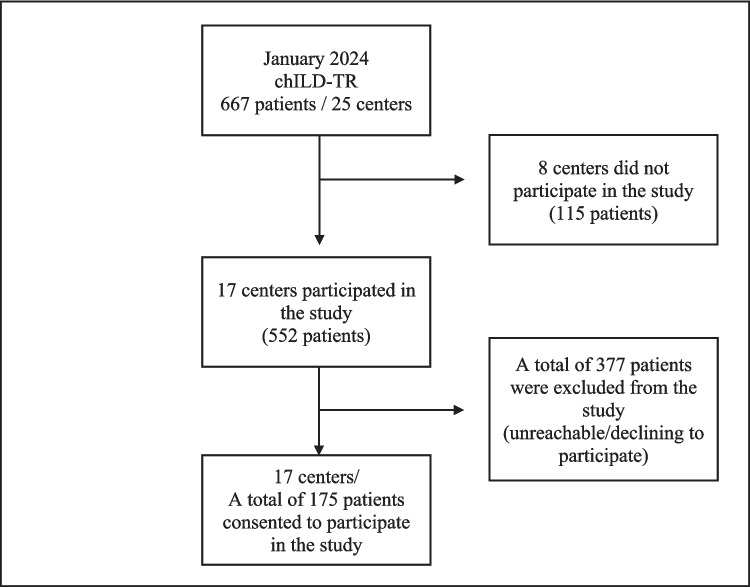
Flow diagram of the study, chILD-TR: childhood interstitial lung disease registry of Turkiye

As of January 2024, the chILD-TR registry included 667 patients from 25 centers. The eight centers (115 patients) did not participate in the study. Seventeen centers participated in the study (*n* = 552), and 337 patients were excluded due to unreachable or declining participation. In those 17 centers, 175 patients consented to participate in the study. The flow diagram of the study is shown in Fig. 1.

Ninety participants (51.4%) were female, and 85 (48.6%) were male. The median current age was 7.7 (1.0–17.8) years, while the age at chILD diagnosis was 1.2 (0.08–16.7) years. The most common presenting symptoms were cough 108 (61.7%), wheezing 62 (35.4%), and dyspnea 59 (33.7%). Seven (4%) patients had a diagnosis of pulmonary hypertension. A total of 28 (16%) patients require respiratory support, 15 (8.5%) use O_2_ with a nasal cannula, 8 (4.6%) of them utilize non-invasive ventilation (NIV), and 5 (2.8%) use mechanical ventilation (MV). Eighteen patients could perform the PFT; the median FEV1% was 69 (25–108), and FVC% was 65 (25–109). Twelve patients could perform the DLCO; the median value was 90 (53–132). All patients’ Fan scores were calculated, and the median value was 2 (1–5). The demographic and clinical data are shown in Table 1.

**Table 1:** Demographic, clinic data, and summary score of all patients

	*n* = 175 (%)
Female Male	90 (51.4) 85 (48.6)
Current age (years)	7.7 (1.0–17.8)
Age at chILD diagnosis (years)	1.2 (0.08–16.7)
Consanguinity between parents	69 (39.4)
Weight z-score^‡^	− 0.93 (− 7.25 to 2.56)
Height z-score^‡^	− 0.72 (− 5.33 to 3.52)
BMI z-score^‡^	− 0.49 (− 8.30 to 2.42)
Symptoms	
Cough Wheezing Dyspnea Feeding difficulties Fever Hemoptysis Cyanosis Chest pain	108 (61.7) 62 (35.4) 59 (33.7) 21 (12) 14 (8) 11 (6.3) 11 (6.3) 4 (2.3)
Pulmonary hypertension	7 (4)
Respiratory support Nasal oxygen NIV MV	28 (16) 15 (8.5) 8 (4.6) 5 (2.8)
Spirometry *n* = 18	
FEV1%^‡^	69 (25–108)
FVC%^‡^	65 (25–109)
FEF_25–75_%^‡^	83 (22–148)
DLCO *n* = 12^‡^	90 (53–132)
Fan score	2 (1–5)
Household income status	
Income is lower than expenditure Income equal to expenditure Income exceeding expenditure	57 (32.6) 80 (45.7) 38 (21.7)
PedsQL Child Self-Report^‡^	
Physical Health Summary Score Psychosocial Health Summary Score Total Score	68.7 (0–100) 68.3 (20–100) 70.1 (26.2–100)
PedsQL Parent Report^‡^	
Physical Health Summary Score Psychosocial Health Summary Score Total Score	68.7 (0–100) 66.6 (5–100) 66.9 (10.7–100)
Family Impact Module^‡^	
Parent Health-Related Quality of Life Score Family Functioning Summary Score Total Score	68.7 (0–100) 69.5 (0–100) 67.6 (0–100)

*BMI* body mass index, *DLCO* diffusion capacity of the lung, *NIV* non-invasive ventilation, *MV* mechanical ventilation

^‡^(median (min–max))

In the PedsQL Child Self-Report, the median Physical Health Summary Score was 68.7 (0–100), the Psychosocial Health Summary Score was 68.3 (20–100), and the Total Score was 70.1 (26.2–100). In PedsQL Parent Report, the median Physical Health Summary Score was 68.7 (0–100), the Psychosocial Health Summary Score was 66.6 (5–100), and the Total Score was 66.4 (10.7–100). In the Family Impact Module, the median Parent HRQOL was 68.7 (0–100), the Family Functioning Summary Score was 69.5 (0–100), and the Total Score was 67.6 (0–100). The PedsQL summary score is shown in Table 1.

Patients were divided into two groups based on their need for respiratory support. The diagnostic age of chILD was 0.75 (0.08–15.5) years in the group with respiratory support and 1.4 (0.08–16.7) years in the group without respiratory support (*p* = 0.046). The group with respiratory support had a higher Fan score, and the median value was 3.5 (1–5) (*p* < 0.001). The median FEV1% value was 25 (25–42) in the group with respiratory support and 69 (43–108) in the group without respiratory support (*p* = 0.008).

The PedsQL Child Self-Report—Physical Health Summary Score, Psychosocial Health Summary Score, and Total Score—and the PedsQL Parent Report—Physical Health Summary Score, Psychosocial Health Summary Score, and Total Score—were lower in the group with respiratory support (*p* < 0.001, *p* = 0.005, *p* < 0.001, *p* = 0.005, *p* = 0.008, *p* = 0.004, respectively). No differences were observed between the groups regarding the Family Impact Module, Parent Health-Related QoL Score, Family Functioning Summary Score, and Total Score (*p* > 0.005).

The comparison of patients with and without respiratory support is shown in Table 2.

**Table 2:** The comparison of patients with and without respiratory support

	With respiratory support (*n* = 28)	Without respiratory support (*n* = 147)	*p*
Female *n* (%) Male *n* (%)	14 (50) 14 (50)	76 (51.7) 71 (48.3)	0.869
Current age (years)^‡^	9.1 (1.08–17.7)	7.4 (1.08–17.8)	0.456
Age at diagnosis of chILD (years)	0.75 (0.08–15.5)	1.4 (0.08–16.7)	**0.046**
Consanguinity between parents	15 (53.5)	54 (36.7)	0.095
Weight z-score^‡^	− 1.4 (− 7.25 to 1.08)	− 0.84 (− 6.81 to 2.56)	0.113
Height z-score^‡^	− 0.78 (− 5.33 to 1.48)	− 0.72 (− 4.99 to 3.52)	0.589
BMI z-score^‡^	− 0.82 (− 8.30 to 2.33)	− 0.44 (− 6.85 to 2.42)	0.427
Number of hospital admissions in the last year *n* (%)^‡^	4 (0–115)	3 (1 − 25)	0.788
Fan score^‡^	3.5 (1–5)	1.5 (1–5)	** < 0.001**
Spirometry	n: 3	n: 15	
FEV1%^‡^	25 (25–42)	69 (43–108)	**0.008**
FVC%^‡^	40 (25–109)	66 (37–102)	0.441
FEF_25–75_%^‡^	45 (23–52)	87 (22–148)	0.101
DLCO^‡^	-	90 (53–132)	-
Household income status			
Income is lower than expenditure Income equal to expenditure Income exceeding expenditure	9 (32.1) 15 (53.6) 4 (14.3)	48 (32.7) 65 (44.2) 34 (23.1)	0.522
PedsQL Child Self-Report^‡^			
Physical Health Summary Score Psychosocial Health Summary Score Total Score	40 (0–72) 60 (25–90) 55 (26–80)	78 (22–100) 72 (20–100) 72(26–100)	** < 0.001** **0.005** ** < 0.001**
PedsQL Parent Report^‡^			
Physical Health Summary Score Psychosocial Health Summary Score Total Score	53 (0–100) 56 (31–81) 56 (23–83)	71 (3–100) 68 (5–100) 69 (10–100)	**0.005** **0.008** **0.004**
Family Impact Module^‡^			
Parent Health-Related QoL-Score Family Functioning Summary Score Total Score	54 (0–100) 52 (0–100) 56 (2–98)	69 (0–100) 72 (0–100) 69 (0–100)	0.057 0.079 0.061

*BMI* body mass index, *DLCO* diffusion capacity of the lung

^‡^(median (min–max))

Bold values indicate statistical significance (p<0.05)

In the DPLD-A group, 6 (22.2%) patients were classified as DPLD-A2, 6 (22.2%) as DPLD-A3, and 15 (56.6%) as DPLD-A4. In the DPLD-B group, 37 (25%) patients were classified as DPLD-B1, 65 (43.9%) as DPLD-B2, 28 (18.9%) as DPLD-B3, 14 (9.5%) as DPLD-B4, and 4 (2.7%) as DPLD-B5. The classification of patients into DPLD-A and DPLD-B subcategories is shown in Table 3.

**Table 3:** Classifications of patients into DPLD-A and DPLD-B subcategories

DPLD-A	*n* = 27 (15.4%)	DPLD-B	*n* = 148 (84.6%)
A2, DPLD-growth abnormalities deficient alveolarization	6 (22.2)	B1, DPLD-related to systemic disease processes	37 (25)
A3, DPLD-infants conditions of undefined etiology	6 (22.2)	B2, DPLD-in the presumed immune-intact host, related to exposures (infectious/non-infectious)	65 (43.9)
A4, DPLD-surfactant dysfunction disorders	15 (56.6)	B3, DPLD-in the immunocompromised or transplanted host	28 (18.9)
		B4, DPLD-related to lung vessels structural processes	14 (9.5)
		B5, DPLD-related to reactive lymphoid lesions	4 (2.7)

Patients were split into two groups, DPLD-A and DPLD-B, without any significant differences in gender or current age (*p* > 0.05). The diagnostic age of chILD was earlier in the DPLD-A group (*p* < 0.001). The weight z-score and BMI z-score were lower in the DPLD-A group (*p* < 0.001, *p* < 0.001). Fan score was detected higher in the DPLD-A group (*p* = 0.026). When groups were compared in terms of household income, income exceeding expenditure was higher in the DPLD-B disorder group (*p* = 0.044). In the Family Impact Module, Parent Health-Related QoL Score and Total Score were lower in the DPLD-B group (*p* = 0.011, *p* = 0.028). The comparison of the DPLD-A and DPLD-B groups is shown in Table 4.

**Table 4:** Comparison of the DPLD-A and DPLD-B groups

	DPLD-A Disorders (*n* = 27)	DPLD-B Disorders (148)	*p*
Female Male	12 (44.4) 15 (55.6)	78 (52.7) 70 (47.3)	0.430
Current age (years)	7.2 (1.08–15.4)	8.5 (1.08–17.8)	0.321
Age at diagnosis (years)	0.08 (0.08–9.25)	1.8 (0.08–16.7)	** < 0.001**
Consanguinity between parents	12 (44.4)	57 (38.5)	0.562
Weight z-score	− 1.97 (− 6.64 to 1.01)	− 0.82 (− 7.25 to 2.56)	** < 0.001**
Height z-score	− 0.67 (− 5.33 to 1.88)	− 0.73 (− 4.99 to 3.52)	0.681
BMI z-score	− 2.12 (− 6.85 to 0.56)	− 0.32 (− 8.30 to 2.42)	** < 0.001**
Number of hospital admissions number in the last year	3.5 (1–22)	4 (0–25)	0.954
Fan score	2.26 ± 1.18	1.76 ± 0.89	**0.026**
Spirometry	***n*: 2**	***n*: 16**	
FEV1%	72 ± 4.9	68 ± 25	0.673
FVC %	65 ± 1.4	68 ± 23	0.888
FEF_25–75_%	96 ± 9	72 ± 22	0.233
DLCO	-	90 (53–132)	-
Household income status			
Income is lower than expenditure Income equal to expenditure Income exceeding expenditure	10 (37) 16 (59.2) 1 (3.7)	47 (31.7) 64 (43.2) 37 (25)	**0.044**
PedsQL Child Self-Report			
Physical Health Summary Score	71 (22–100)	65 (0–100)	0.369
Psychosocial Health Summary Score	71 (41–100)	67 (20–100)	0.608
Total Score	69 (40–100)	70 (26–100)	0.537
PedsQL Parent Report			
Physical Health Summary Score	68 (28–100)	68 (0–100)	0.605
Psychosocial Health Summary Score	65 (40–96)	66 (5–100)	0.382
Total Score	63 (39–97)	66 (10–100)	0.525
Family Impact Module			
Parent Health-Related QoL-Score	85 (38–100)	65 (0–100)	**0.011**
Family Functioning Summary Score	75 (30–100)	69 (0–100)	0.282
Total Score	80 (38–98)	66 (0–100)	**0.028**

*BMI* body mass index, *DLCO* diffusion capacity of the lung, *DPLD* diffuse progressive lung disease, *PFT* pulmonary function test

Bold values indicate statistical significance (p <0.05)

There was a weak negative correlation between the chILD diagnosis age and the Psychosocial Health Summary Score and Total Score (*p* = 0.026, *r* = − 0.204, *p* = 0.043, *r* = − 0.186). There was a strong positive correlation between the FEV1% value and the Physical Health Summary Score and the Total Score (*p* = 0.007, *r* = 0.608, *p* = 0.008, *r* = 0.607). Also, a moderate positive correlation was found between FVC% and Physical Health Summary Score (*p* = 0.047, *r* = 0.473).

There was a moderate positive correlation found between the FEV1% and Physical Health Summary Score, Psychosocial Health Summary Score, and a strong positive correlation between the Total Score (*p* = 0.025, *r* = 0.527, *p* = 0.032, *r* = 0.522, *p* = 0.009, *r* = 0.612). There was a moderate positive correlation between the FVC% and Physical Health Summary Score and Total Score (*p* = 0.025, *r* = 0.525, *p* = 0.028, *r* = 0.531). Also, there was a weak negative correlation between Fan score and Physical Health Summary Score (*p* < 0.001, *r* = − 0.363), a moderate negative correlation between Fan score and Psychosocial Health Summary Score, and Total Score (*p* < 0.001, *r* = − 0.420, *p* < 0.001, *r* = − 0.427).

There was a weak negative correlation between the Fan score and Parent Health Related QoL Score, Family Functioning Summary Score, and the Total Score (*p* < 0.001, *r* = − 0.302, *p* = 0.010, *r* = − 0.217, *p* = 0.001, *r* = − 0.291). No correlation was detected between Family Impact Module—Parent Health Related QoL Score, Family Functioning Summary Score, and Total Score—and other demographic and clinical parameters (*p* > 0.05).

The correlation between demographic and clinical parameters and the PedsQL Child Self-Report score, PedsQL Parent Report, and Family Impact Module score is shown in Table 5.

**Table 5:** Correlation of clinical parameters and PedsQL Child Self-Report score, PedsQL Parent Report, and Family Impact Module

	PedsQL Child Self-Report						PedsQL Parent Report						Family Impact Module					
	Physical Health Summary Score		Psychosocial Health Summary Score		Total Score		Physical Health Summary Score		Psychosocial Health Summary Score		Total Score		Parent Health Related QoL Score		Family Functioning Summary Score		Total Score	
	*p*	*r*	*p*	*r*	*p*	*r*	*p*	*r*	*p*	*r*	*p*	*r*	*p*	*r*	*p*	*r*	*p*	*r*
Current age	0.006	− 0.247	0.020	− 0.214	0.013	− 0.227	0.089	− 0.136	**0.042**	** − 0.175**	0.100	− 0.142	0.569	− 0.047	0.737	− 0.028	0.555	− 0.050
Age at diagnosis	0.075	− 0.161	**0.026**	** − 0.204**	**0.043**	** − 0.186**	0.785	− 0.022	0.622	− 0.043	0.865	0.015	0.636	− 0.039	0.460	− 0.062	0.369	− 0.076
Weight z-score	0.720	0.033	0.927	− 0.008	0.998	0.000	0.170	0.111	0.406	0.072	0.339	0.084	0.797	0.022	0.773	0.025	0.910	0.010
Height z-score	0.733	0.032	0.994	− 0.001	0.973	0.003	0.301	0.086	0.387	0.078	0.299	0.094	0.823	− 0.019	0.872	0.014	0.872	0.014
BMI z-score	0.381	0.080	0.762	0.028	0.612	0.047	0.141	0.128	0.137	0.130	0.308	0.086	0.317	0.084	0.712	0.032	0.545	0.052
HA*	0.169	− 0.258	0.889	0.028	0.592	− 0.106	0.905	0.020	0.239	− 0.218	0.331	− 0.181	0.647	0.083	0.610	0.094	0.648	0.084
Fan score	** < 0.001**	** − 0.536**	** < 0.001**	** − 0.447**	** < 0.001**	** − 0.540**	** < 0.001**	** − 0.363**	** < 0.001**	** − 0.420**	** < 0.001**	** − 0.427**	** < 0.001**	** − 0.302**	**0.010**	** − 0.217**	**0.001**	** − 0.291**
FEV1%	**0.007**	**0.608**	0.099	0.401	**0.008**	**0.607**	**0.025**	**0.527**	**0.032**	**0.522**	**0.009**	**0.612**	0.098	0.443	0.990	0.004	0.446	0.213
FVC%	**0.047**	**0.473**	0.621	0.125	0.096	0.405	**0.025**	**0.525**	0.073	0.446	**0.028**	**0.531**	0.540	0.172	0.491	0.193	0.616	0.141
FEF_25–75_%	0.105	0.406	0.191	0.334	0.080	0.436	0.078	0.354	0.240	0.331	0.097	0.429	0.342	0.275	0.180	− 0.380	0.737	− 0.099
DLCO	0.165	0.476	0.090	0.564	0.162	0.479	0.130	0.485	0.385	0.291	0.326	0.327	0.412	0.293	0.751	0.115	0.855	0.067
Mother age	0.753	0.029	0.443	− 0.072	0.944	0.007	0.961	− 0.004	0.962	0.004	0.641	0.004	0.374	0.074	0.674	− 0.036	0.818	− 0.020
Father age	0.342	0.089	0.960	− 0.005	0.279	0.103	0.760	− 0.025	0.794	− 0.023	0.961	0.004	0.539	0.052	0.933	0.007	0.773	0.025

*BMI* body mass index, *DLCO* diffusion capacity of the lung

*HA: number of hospital admissions last year

Bold values indicate statistical significance (p <0.05)

## Discussion

Assessing the HRQoL life of the child and their family is essential in all chronic diseases; however, it becomes particularly crucial in rare, poorly understood, complex conditions such as chILD, where the diagnosis and disease management are challenging and multidimensional impacts are often overlooked. This multicenter national study provides the first comprehensive assessment of HRQoL and the family impact on children diagnosed with chILD in Turkiye. This study demonstrated that lung function and Fan score strongly correlate with HRQoL, emphasizing the effects of disease severity on physical and psychosocial well-being.

The World Health Organization defines QoL as “an individual’s perception of their position in life in the context of the culture and value systems, in which they live and in relation to their goals, expectations, standards, and concerns.” It is a complex concept shaped by an individual’s physical health, mental state, personal beliefs, social connections, and their interaction with essential elements of their environment [6, 17]. In Turkiye, this issue becomes even more important due to the high prevalence of consanguineous marriages and the relatively frequent occurrence of rare diseases.

Importantly, objective pulmonary function parameters, especially FEV1, are among the most widely accepted indicators of pulmonary function and disease progression in chronic respiratory conditions [18]. They reflect airway obstruction and ventilatory capacity and are closely linked to morbidity and mortality, particularly in diseases such as cystic fibrosis, bronchopulmonary dysplasia, and chILD. A decline in FEV1 is frequently associated with increased symptom burden, exercise limitation, and healthcare utilization. Additionally, the Fan score is a clinical scoring system designed to assess the severity of diseases in ILD. It quantitatively evaluates disease burden based on oxygen requirements and the presence of pulmonary hypertension to measure overall disease severity [14]. In our study, both FEV1 and Fan scores demonstrated strong, consistent correlations with HRQoL measures reported by children and their caregivers, particularly within the physical and psychosocial domains. Lower FEV1 and higher Fan scores were associated with diminished PedsQL scores, underscoring the clinical and patient-centered relevance of these measures. These findings highlight the value of incorporating both HRQoL and Fan score routine clinical assessments, which correlate well with subjective patient and caregiver experiences. They can serve as practical guides for monitoring disease progression, evaluating treatment response, and acting as surrogate markers for multidimensional disease burden. In settings where spirometry is not feasible, such as in very young or severely ill patients, HRQoL assessments and the Fan score may offer practical, complementary tools to evaluate disease impact, tailor interventions, and guide comprehensive care in chILD.

Niemitz et al. [3], in a study conducted within the framework of the chILD-European registry, developed and validated the chILD-QoL questionnaire. They found that lower QoL scores were significantly associated with more severe disease features, such as dyspnea, tachypnea, and a poorer overall clinical course. A study by Lauby et al. [5] included 78 children from 13 centers as part of the French national chILD registry (e-RespiRare). They compared QoL scores of children with chILD to those of age-matched healthy controls and found significantly lower scores in the chILD group. Poorer QoL was independently associated with higher Fan severity scores, extrapulmonary involvement, nutritional support, and the use of multiple oral medications. Similarly, our findings support this, further demonstrating that disease severity and treatment-related factors substantially affect HRQoL in children with chILD. These findings highlight the substantial physical and psychosocial burden of chILD and underscore the strong connection between disease severity and HRQoL as perceived by patients or caregivers in pediatric chILD populations, emphasizing the importance of routine QoL assessments during clinical follow-up.

Previous studies have demonstrated a connection between the decline and deterioration of HRQoL in children on MV [19]. Laubry et al. [5] also demonstrated that oxygen supplementation is associated with a decrease in assessing child patients’ HRQoL. This finding is consistent with our results, which indicate that child-reported and parent-reported HRQoL scores are significantly lower in children who require supplemental oxygen therapy. Physical health, psychosocial functioning, and total scores were notably worse in this group. This aligns with previous findings indicating that oxygen dependence is a strong marker of disease severity in pediatric pulmonary conditions. The lower HRQoL scores observed in our cohort suggest that oxygen therapy not only indicates more advanced lung disease but also reflects the disease's broader impact on daily life, mobility, independence, and emotional well-being. Interventions targeting oxygen-dependent children, such as home-based rehabilitation, psychosocial support, and nutritional optimization, may help reduce the adverse impact on QoL. Additionally, despite expectations, family impact scores did not vary based on respiratory support status. This may indicate that families have developed effective coping strategies and benefit from strong cultural or social support systems that lessen the psychosocial effects of the disease.

The care process for children with chronic illnesses affects not only the patient but also the overall QoL for the entire family. Previous studies using the PedsQL™ Family Impact Module have shown that such conditions negatively impact parents’ physical, emotional, and social functioning, straining daily routines and family dynamics. Additionally, home-based care has been reported to place greater stress on caregivers due to the ongoing responsibilities and psychological demands involved [8, 20]. In our study, children receiving respiratory support had significantly lower PedsQL scores, as reported by both the child and the parent, indicating poorer HRQoL associated with greater clinical severity. However, no significant difference was observed in Family Impact Module scores between families of children who used oxygen and those who did not. This finding contrasts with earlier literature suggesting that higher disease severity in children leads to greater family burden. One possible explanation is that families may develop adaptive mechanisms and strategies over time, helping them maintain family functioning despite the child’s deteriorating clinical status. Alternatively, cultural dynamics, close kinship relations, and evolving social support systems, which are relatively common in our society, may buffer the perceived impact of the disease on family life. Further qualitative research is warranted to gain a deeper understanding of these contextual factors.

In this study, significant differences were observed between the DPLD-A and DPLD-B groups in terms of clinical parameters and Family Impact Module assessments. Patients in the DPLD-A group had lower weight and BMI z-scores and higher Fan scores, indicating a more severe clinical status. Also, the younger age profile and disease characteristics of the DPLD-A group may partly contribute to these differences in growth z-scores. It is known that early-onset DPLD is often associated with prolonged oxygen dependency, recurrent infections, and feeding difficulties, all of which may hinder optimal growth despite appropriate nutritional support. Therefore, the lower anthropometric scores in this group likely reflect a combination of disease severity and early-life complications rather than isolated malnutrition. Interestingly, despite these differences, no statistically significant difference in PedsQL was reported by either the children or their parents. This finding suggests that perceived QoL is not solely determined by clinical severity but may also be influenced by factors such as individual adaptation, coping strategies, and psychosocial support. Notably, Family Impact Module scores were significantly lower in the DPLD-B group, indicating greater disruption to family functioning and caregiver burden. One possible explanation is the higher age at diagnosis noted in the DPLD-B group, which may have delayed the adaptation process and increased the psychosocial strain on the family. Furthermore, families in the DPLD-B group had higher socioeconomic status, which may be linked to heightened expectations regarding their child’s health and functioning, thereby amplifying the perceived negative impact of the disease.

The cross-sectional design and the small number of patients who underwent PFT and DLCO measurements are significant limitations of this study, as they restrict the generalizability of the physiologic correlation findings. Another limitation is that younger children may have had difficulty understanding the PedsQL items; however, assistance from parents or trained healthcare staff was provided when needed to ensure accurate understanding and completion of the questionnaire.

In conclusion, this study emphasizes the significant impact of chILD on children’s physical and psychosocial health, particularly among those experiencing severe disease and requiring respiratory support. While clinical parameters are associated with HRQoL, the effects on families appear to be influenced by more complex factors, such as socioeconomic conditions and potential cultural resilience. Future research should include longitudinal HRQoL assessments and investigate targeted interventions to improve outcomes for children and their families affected by chILD. These findings underscore the necessity for multidisciplinary care and psychosocial support to enhance the QoL for children with chILD and their families.

## Data Availability

The data supporting the findings of this study can be obtained from the corresponding author upon a reasonable request.
